# Modifications of cysteine residues in the transmembrane and cytoplasmic domains of a recombinant hemagglutinin protein prevent cross-linked multimer formation and potency loss

**DOI:** 10.1186/s12896-014-0111-y

**Published:** 2014-12-24

**Authors:** Kathleen M Holtz, Pamela S Robinson, Erin E Matthews, Yoshifumi Hashimoto, Clifton E McPherson, Nikolai Khramtsov, Michael J Reifler, Jamal Meghrous, David G Rhodes, Manon M Cox, Indresh K Srivastava

**Affiliations:** Protein Sciences Corporation 1000 Research Parkway, Meriden, CT 06450 USA

**Keywords:** Hemagglutinin, Influenza, Vaccine, Potency, Protein cross-linking, Protein stability, Antigen, Cysteine

## Abstract

**Background:**

Recombinant hemagglutinin (rHA) is the active component in Flublok®; a trivalent influenza vaccine produced using the baculovirus expression vector system (BEVS). HA is a membrane bound homotrimer in the influenza virus envelope, and the purified rHA protein assembles into higher order rosette structures in the final formulation of the vaccine. During purification and storage of the rHA, disulfide mediated cross-linking of the trimers within the rosette occurs and results in reduced potency. Potency is measured by the Single Radial Immuno-diffusion (SRID) assay to determine the amount of HA that has the correct antigenic form.

**Results:**

The five cysteine residues in the transmembrane (TM) and cytoplasmic (CT) domains of the rHA protein from the H3 A/Perth/16/2009 human influenza strain have been substituted to alanine and/or serine residues to produce three different site directed variants (SDVs). These SDVs have been evaluated to determine the impact of the TM and CT cysteines on potency, cross-linking, and the biochemical and biophysical properties of the rHA. Modification of these cysteine residues prevents disulfide bond cross-linking in the TM and CT, and the resulting rHA maintains potency for at least 12 months at 25°C. The strategy of substituting TM and CT cysteines to prevent potency loss has been successfully applied to another H3 rHA protein (from the A/Texas/50/2012 influenza strain) further demonstrating the utility of the approach.

**Conclusion:**

rHA potency can be maintained by preventing non-specific disulfide bonding and cross-linked multimer formation. Substitution of carboxy terminal cysteines is an alternative to using reducing agents, and permits room temperature storage of the vaccine.

## Background

Licensed, seasonal influenza vaccines available in the United States include trivalent and quadrivalent inactivated vaccines and a live attenuated influenza vaccine produced in embryonated chicken eggs [1,2], a cell culture based trivalent vaccine produced in Madin Darby Canine Kidney (MDCK) cells [3,4], and most recently a recombinant trivalent vaccine (Flublok) produced using the baculovirus-insect cell system [5-7]. Flublok vaccine has several distinct advantages over other flu vaccines including high purity of the HA protein, and absence of antibiotics, preservatives, gelatin, and egg proteins.

HA, the most abundant and immunogenic surface antigen of the influenza virus, is responsible for mediating viral attachment by binding to sialic acid residues on the host cell surface, and for fusing the viral envelope to the host cell membrane [8]. The HA protein is a homotrimer extending 135 Å from the viral membrane, and consists of a stem-like region formed by three helices, one from each monomer, and a globular head domain containing antigenic epitopes. These two domains form the ectodomain which has previously been solubilized by bromelain cleavage to determine its crystal structure [9-11]. The TM domain sequence has a propensity to form alpha-helical oligomers in model systems [12-14] and this tendency may extend to the alpha-helices of the stem region [15]; however, the structure of this domain, as well as the conformation of C-terminal amino acids of the CT, has not been determined.

Flublok includes three rHA proteins (full length without signal peptide) that are highly purified (≥90%) using our universal purification process, and are updated according to the annual influenza strain selection process [5]. By comparison, the whole virus vaccines produced using the traditional egg based system are chemically inactivated with either formaldehyde or beta-propiolactone (BPL) and partially purified by either column chromatography or sucrose gradient ultracentrifugation and filtration [16-18]. Split and subunit vaccines produced using both the egg and cell culture systems include a detergent extraction step, as well as an additional sucrose gradient or alternative purification step, to further reduce the lipid and contaminating protein content [16-18]. Despite considerable variation in the manufacturing processes and purity, the quantification or potency of the HA proteins produced either in eggs or in BEVS is determined using the SRID assay [19,20]. The SRID based potency assay, in use since 1978, is required to standardize HA content in inactivated licensed flu vaccines by the FDA. The SRID assay uses strain specific anti-HA antibodies to quantify HA trimer in the presence of the surfactant Zwittergent 3–14.

**Figure 1:**
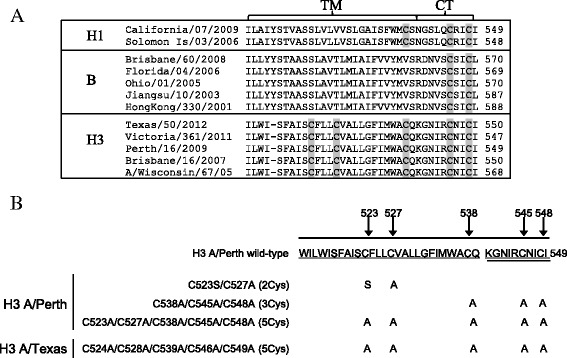
**A and B Amino acid sequence of the TM and CT domains. A**. Shown is a sequence alignment of the transmembrane (TM) and cytoplasmic tail (CT) domains in the hemagglutinin protein. Protein sequences from different human influenza strains belonging to the H1, B, and H3 subtypes have been included and the cysteine residues highlighted. **B**. The amino acid sequences of the wild-type and site directed variants of H3 A/Perth and H3 A/Texas rHA are shown. The position of the cysteine residues and the modifications are indicated. The sequences for the TM and CT domains are underlined and double underlined, respectively.

Purified wild-type rHA proteins, particularly H3 rHAs, show an apparent initial loss of potency in the SRID assay before leveling off within typically four weeks after production. In the case of H3 rHA from the A/Perth/16/2009 influenza strain included in the 2010–2011, and 2011–2012 Flublok vaccines, this apparent initial loss of potency is as high as 40% and is correlated with an increase in disulfide bond cross-linking, and the oxidation of at least one conserved cysteine residue at position 549 of the primary sequence in the CT domain [21]. HA proteins derived from human influenza viruses can contain 2–5 cysteine residues in the TM and CT domains depending on the subtype (Figure 1A) in addition to twelve conserved cysteine residues located in the ectodomain which form six sets of disulfide bonds based on X-ray crystal structure analysis and are required for proper folding [22-24]. These cysteine residues have been implicated in membrane fusion, viral assembly and replication [25-28]. More recent studies have examined the importance of the TM and CT cysteine residues on thermal stability, heterotypic protection in mice, and other virological features [29-32]. In the context of influenza viruses, these cysteines in HA proteins are modified with palmitic acid which may be important for biological function. While the baculovirus-insect cell system supports palmitoylation [33,34], such reversible, post-translational modifications may be incomplete, as the available data indicate that at least one of the two C-terminal cysteine is partially free and undergoes oxidation [21].

In this study, we evaluated the role of the TM and CT cysteine residues on the thermal stability as determined by DSF, functional stability as determined by potency, antigenicity, and tertiary and quaternary structure of the H3 rHA protein from the A/Perth/16/2011(H3 A/Perth) influenza strain. All or a portion of the TM and CT cysteine residues were substituted with either serine or alanine to produce SDV rHA proteins. Our results demonstrate that this cysteine substitution strategy can prevent an apparent potency loss associated with disulfide mediated cross-linking, but does not impact the expression, purification, folding, or thermal stability of the molecule. Hemagglutination inhibition, an assay used to determine the antigenic identity of the vaccine viruses, has been used to show that the SDV rHAs are antigenically similar to wild type rHA, and that the cysteine substitution strategy does not impact the antigen specific antibody responses in mice upon immunization. The SDV rHAs are presented to the immune system in the same quaternary structural form as the wild-type rHA based on the comparability of the rosette structure as determined by negative stain electron microscopy data. Finally, the cysteine substitution strategy has been applied to another H3 rHA derived from the A/Texas/50/2012 (H3 A/Texas) influenza strain with comparable results demonstrating that the approach can be applied to address in vitro potency loss in H3 rHAs caused by the formation of non-native disulfide bonds.

## Results

### Comparable expression and purification of the wild-type and site directed variants

To examine the role of the cysteine residues located in the TM and CT of the H3 A/Perth rHA protein, alanine or serine substitutions were introduced into the wild-type gene by site directed mutagenesis. Three different SDVs were produced having two, three, and five cysteine substitutions. They are referred to by the number of cysteine substitutions or as 2Cys, 3Cys and 5Cys, respectively (Figure 1B). Alanine was selected based on its small size, similar hydrophobicity, and lack of chemistry including hydrogen bonding. A cysteine to serine substitution was made at position 523 in the 2Cys SDV based on amino acid sequence alignments consistently showing a serine at the equivalent position of HA proteins from H1 and B subtypes (Figure 1A). As a control in this study, a pair of conserved cysteine residues in the ectodomain known to form a disulfide bond, C64 and C76, was disrupted by substitution with serine residues. Finally, to verify the relative potency findings obtained with the H3 A/Perth 5Cys SDV rHA in this study, the same strategy was used to replace all five TM/CT cysteine residues with alanine residues in the H3 A/Texas rHA protein (Figure 1A and B).

The universal purification process for the wild-type rHA was applied to the SDV rHA proteins without modification. The expression level for the control SDV replacing the two conserved and paired cysteines in the ectodomain was too low to purify (Table 1). The potency of the soluble rHA in the supernatant fraction was determined in the SRID assay and was used to establish the starting yield. The final yield after purification was determined using the total protein results obtained by the BCA assay corrected for the rHA purity determined by SDS-PAGE. The results provided in Table 1 show that the starting and purified yields for the 2Cys, 3Cys, and 5Cys SDV rHA proteins relative to the wild-type rHA were equivalent or better compared to the wild-type rHA protein.

**Table 1:** **Starting and final purified yields for rHA proteins**

**rHA**	**Starting yield**	**Purified yield**
	**% of wild-type**	**% of wild-type**
Wild-type	100	100
Control SDV*	13	Not Done
2Cys	97	120
3Cys	118	199
5Cys	138	136

*SDV contains modifications C64S, C76S in the ectodomain of the protein.

**Figure 2:**
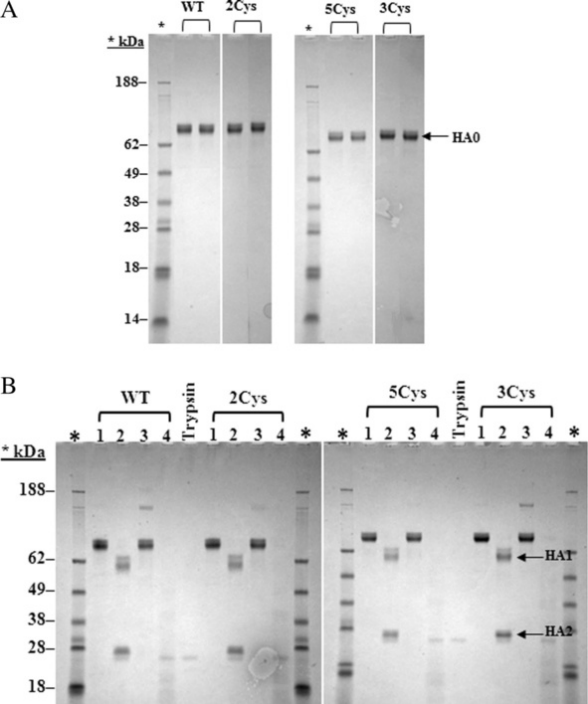
**A and B SDS-PAGE purity and trypsin resistance gels for the H3 rHA proteins. A**. Each rHA protein was loaded in duplicate to give 1 μg total protein per lane. The samples were separated using 4-12% gradient Nu-PAGE gels and stained with Coomassie Blue. Molecular weights of the proteins standards are shown. **B**. Shown are the reducing SDS-PAGE gels from the trypsin resistance assay. Each H3 rHA protein was analyzed neat (Lane 1), after trypsin treatment (Lane 2), after heat treatment (Lane 3), and after heat and trypsin treatment (Lane 4). The trypsin enzyme is loaded as a control and molecular weights of the protein standards are shown. HA1 and HA2 are indicated by arrows.

Purity of rHA was determined by reducing SDS-PAGE. The full length rHA proteins migrates as monomers (HA0) having an approximate molecular weight of 62 kDa (Figure 2A) under denaturing and reducing conditions. The purity of the rHA proteins exceeds 99%.

### rHA folding and activity were unaffected by substitutions of the TM/CT cysteines

The trypsin resistance assay provides a qualitative assessment of the folded state of the rHA protein. All the trypsin recognition sites are masked in the correctly folded full-length, HA trimer with the exception of one site located toward the end of the stem region. As a result, properly folded rHA protein is cleaved by trypsin into HA1 and HA2, while denatured or mis-folded rHA degrades in the presence of trypsin. Each rHA was trypsin treated with and without heat denaturation and analyzed by reducing SDS-PAGE alongside the untreated rHA with and without heat denaturation (Figure 2B). All rHAs were resistant to trypsin digestion and the trypsin resistance profiles obtained for the SDV rHAs were comparable to that of the wild-type rHA. In the presence of trypsin, the monomeric rHA band (~60 kDa) was cleaved into HA1 and HA2 that migrated as ~55 kDa and ~25 kDa proteins, respectively (Figure 2B, lane 2). Boiling the rHA sample prior to trypsin treatment denatured the protein and resulted in digestion of the rHA by trypsin (Figure 2B, lane 4).

**Figure 3:**
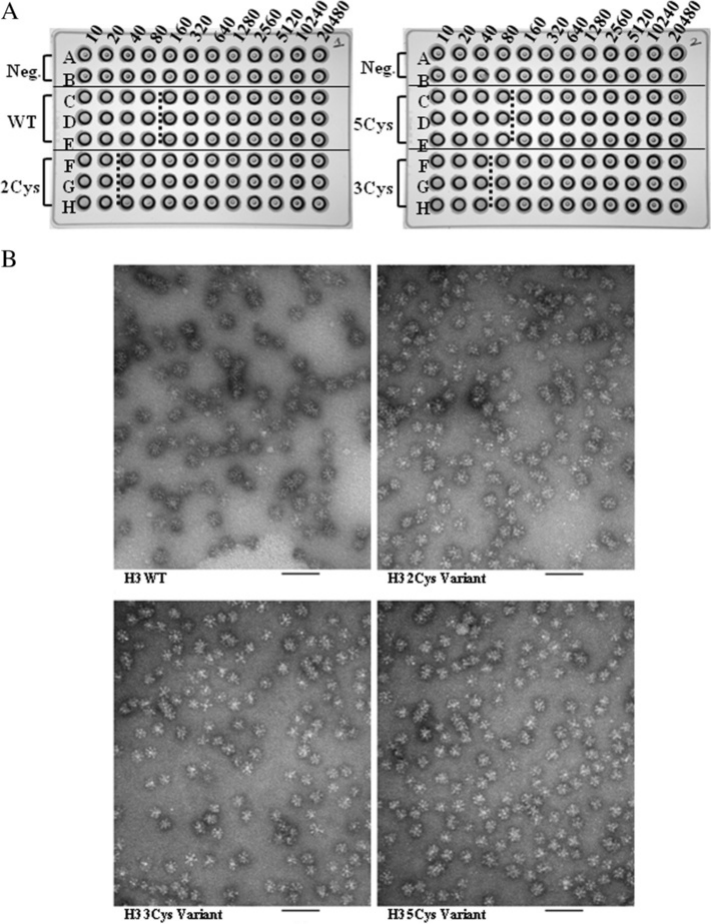
**A and B Hemagglutination activity and negative stain electron microscopy of the H3 rHA proteins. A**. The hemagglutination assay was performed in 96-well u-bottom plates. The rHA proteins were assayed in triplicate at an initial concentration of 1 μg/mL loaded in the left-most lane. Two-fold serially dilutions were performed across the plate in PBS, and equal volume 0.5% guinea pig red blood cells added. Endpoints are denoted by dotted lines. **B**. The rHA proteins were stored at 25°C for 2.5 month at the time of EM analysis. The bar represents 100 nm.

To determine whether the SDV rHAs contain functionally active forms of the protein, they were evaluated in a hemagglutination assay using the wild-type H3 rHA protein as control. Agglutination of red blood cells requires the HA trimers to be organized in higher order (rosette) structures. The globular head of the rHA trimer forms a receptor binding site for terminal sialic acids on red blood cells. The arrangement of trimers in a rosette structure results in a lattice like structure of the bound cells which do not settle out. Unbound cells settle out and produce a button like or halo pattern of cells as observed in negative controls (Figure 3A). The HA activity of the H3 SDV rHAs were within 2–4 -fold of the HA activity of the wild-type H3 rHA (Table 2).

**Table 2:** **Hemagglutination activity results for wild-type and SDV rHAs**

**rHA protein**	**HA activity**
	**Units/μg**
Wild-Type	160
2Cys	40
3Cys	80
5Cys	160

### Particle size is unaffected by cysteine substitutions

The particle size of the H3 rHA proteins was determined by dynamic light scattering (DLS), and the results for the volume mean diameter are provided in Table 3. The mean volume diameter for the wild-type H3 rHA, and the 5Cys and 3Cys SDV H3 rHAs are comparable (average 37.2 ± 2.1). In contrast, the mean volume diameter of the 2Cys SDV H3 rHA was consistently larger in size (average 50.5 ± 1.8) and may be due to the alternative cysteine to serine substitution in this SDV. Despite this difference, the mean volume diameter of the particles observed for the wild-type and SDV H3 rHAs by DLS (range 30–50 nm) was consistent with the size of rosettes visible by EM [35,36].

**Table 3:** **Volume mean diameter by DLS for wild-type and Cys SDV H3 rHAs**

**Sample name**	**Volume mean diameter (nm)**			
	**Day 0**	**Day 7**	**Day 28**	**Average**
Wild-type	38.9	39.2	39.6	39.2 ± 0.4
5Cys	36.5	36.3	38.8	37.2 ± 1.4
3Cys	35.5	33.5	36.3	35.1 ± 1.4
2Cys	52.6	49.7	49.3	50.5 ± 1.8

The morphology of the rosettes was determined by electron microscopy (EM). For this analysis, aliquots of rHA protein samples stored for 2.5 month at 25°C were submitted to Paragon Bioservices for electron microscopy. Representative EM images are shown in Figure 3B. Rosette size, estimated to be approximately 30–50 nm, and spike densities were similar in the EM images for the wild-type H3 rHA and the SDVs.

**Figure 4:**
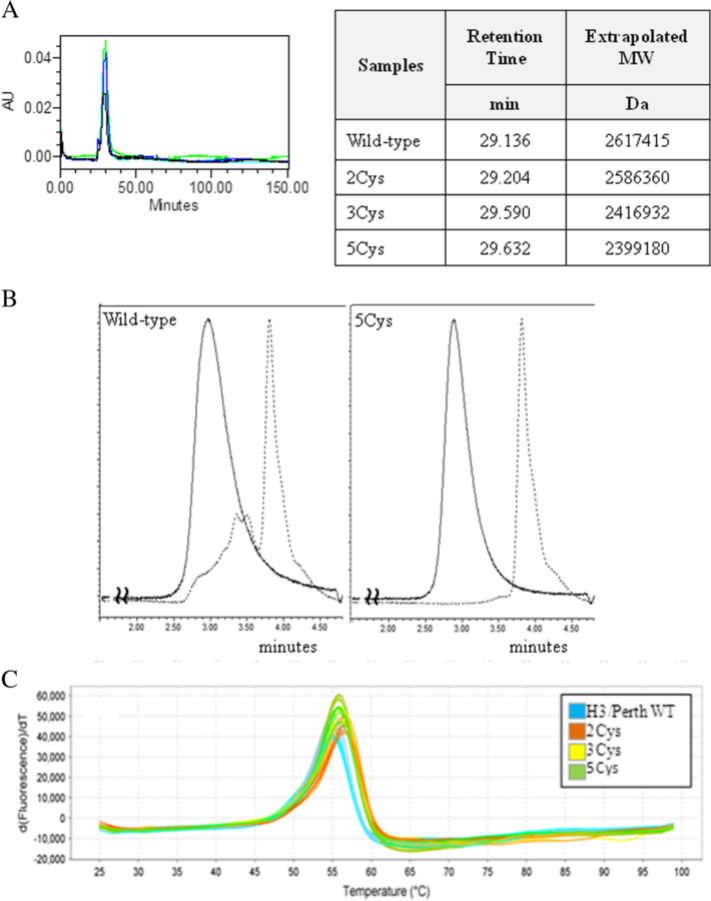
**A**-**4C SEC-HPLC elution profiles, SEC-UPLC elution profiles, and thermal denaturation curves using DSF for the rHA proteins. A**. The SEC-HPLC elution profiles for the wild-type and SDV rHA proteins overlay. The retention times and extrapolated MWs are similar for the rHA proteins. The # of trimers per rosette was estimated using the extrapolated MW and a trimer MW of ~210 kDa. **B**. The SEC-UPLC elution profiles before (solid lines) and after (dotted lines) Zwittergent 3–14 pre-treatment are shown in for the wild-type (left) and 5Cys SDV rHA (right). **C**. The melting temperature (T_m_) was determined by DSF, and the second derivative thermal denaturation curves are shown for each rHA.

SEC was performed to determine the homogeneity of the preparations. The SEC elution profiles were similar for the rHAs (Figure 4A). The retention time of the primary peak (average 29.39 ± 0.26) and the extrapolated MWs (average 2,505 kDa ± 113 kDa) were similar for all the rHAs. In addition, the extrapolated numbers of trimers per rosette for the wild-type rHA and SDV rHAs remained constant [11,12] suggesting that the cysteine substitution did not affect the assembly of the trimers into rosettes.

Both the wild type H3 rHA and 5 Cys SDV rHA were analyzed by SEC UPLC before and after treatment with 1% Zwittergent 3–14, a surfactant which converts rHA rosettes into the trimeric form. In the absence of Zwittergent treatment, the wild-type and 5Cys SDV rHAs gave a single broad peak by SEC with a retention time of approximately 3 minutes (Figure 4B). Pre-treatment of a 5Cys SDV rHA with 1% Zwittergent 3–14 prior to SEC-UPLC analysis shifted the broad peak to a narrower peak having a retention time of 3.8 minutes. In contrast, the SEC-UPLC profile for the wild-type rHA pre-treated with 1% Zwittergent 3–14 resulted in multiple peaks with retention times between 3 and 3.8 minutes. These results support the presence of disulfide cross-linked multimers that are not disrupted by the Zwittergent treatment in the wild-type rHA. In contrast, the 5Cys SDV rHA does not have disulfide mediated cross-linked structures and completely shifts to a trimeric conformation upon treatment with 1% Zwittergent 3–14.

### Thermal stability of rHA is unaffected by cysteine substitutions in the TM and CT domains

The wild-type H3 rHA and Cys SDV H3 rHAs were analyzed from 25°C to 99°C by DSF in the presence of a molecular rotor dye (ProteoStat, Enzo Life Sciences) to determine thermal stability. Fluorescence intensity was monitored as a function of temperature and a single, large cooperative unfolding was observed for each protein (Figure 4C). These data showed that T_m_s of SDV rHA were all similar to the wild-type H3 rHA (Table 4), and these differences in the T_m_s are not statistically significant. Since the thermal stability of the SDVs as determined by DSF was quite similar to the wild-type rHA, this data further supports that the effect of cysteine substitutions on the overall structure of the rHA is minimal.

**Table 4:** **Melting temperatures for wild-type and Cys SDV H3 rHAs using DSF**

**Protein**	**TM mean (n = 5)**	**Standard deviation**
Wild-type	55.08	0
5Cys	55.82	0
3Cys	56.27	0.17
2Cys	56.71	0.20

### Cysteine modifications affect potency loss, cross-linking, and hydrophobicity

**Figure 5:**
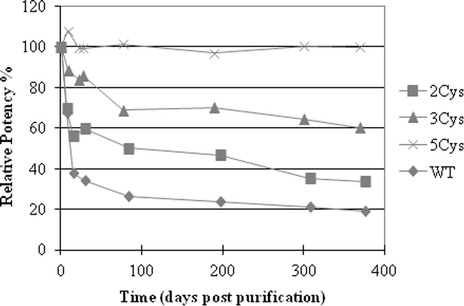
**Potency determined by SRID for the wild-type and SDV rHA proteins stored at 25°C.** The rHA proteins were placed at 25°C storage and the potency measured at different time points by SRID. Shown are the potency results plotted relative to the day 0 value for each H3 A/Perth rHA in the study.

The wild-type H3 rHA and Cys SDV H3 rHAs were stored at 25°C and potency by SRID and cross-linking by SDS-PAGE were monitored. The potency was determined by SRID on the day of final purification (day 0), day 7, day 14 or 21, and day 28, and after approximately 2.5, 6, 9, and 12 months. The results were plotted relative to the day 0 results for wild-type rHA, as well as, for each H3 SDV rHA (Figure 5). The relative potencies of the 5Cys and 3Cys SDV H3 rHAs showed little change over time and were significantly improved compared to the wild-type and 2Cys SDV H3 rHA proteins. Based on the results, the loss in potency decreases with the number of cysteine substitutions in the order WT > 2Cys > 3Cys > 5Cys.

**Figure 6:**
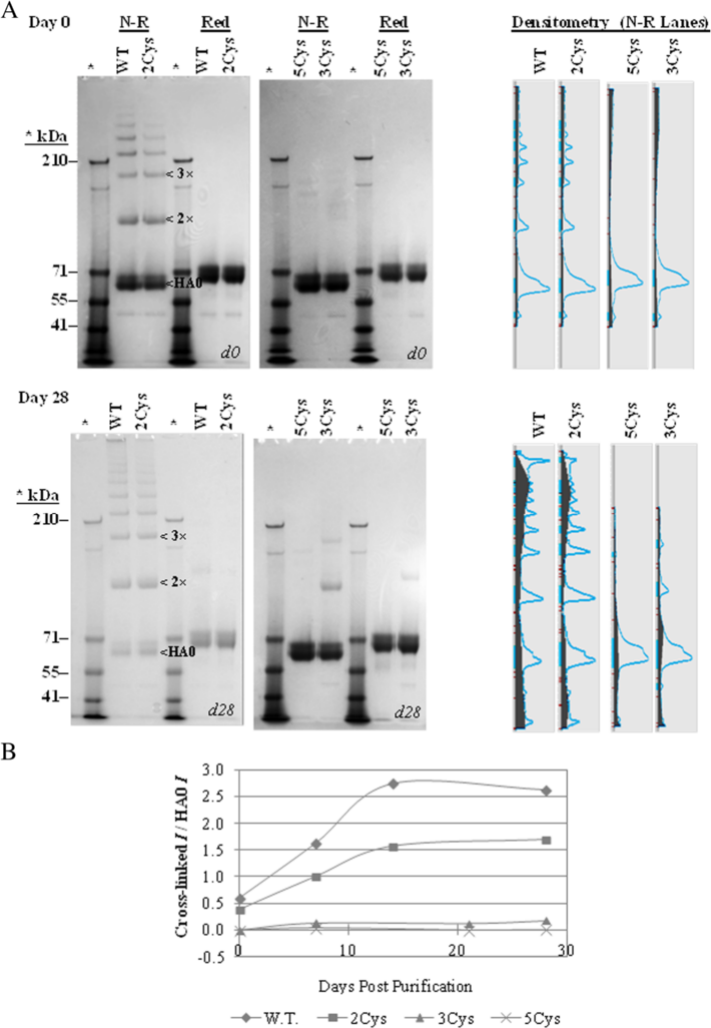
**A and B Non-reducing and reducing SDS-PAGE gels and densitometry for rHA proteins on days 0 and 28. A**. Each rHA (~2.5 μg) was analyzed by SDS-PAGE under non-reducing (N-R) and reducing (Red) conditions on days 0, 7, 14 or 21, and 28. Shown are gels obtained on day 0 (top) and on day 28 (bottom). Densitometry was performed on the non-reducing lanes on the left-hand-side of each gel using the Carestream software program, and the corresponding intensity profiles are shown on the right. **B**. The intensities of individual bands in the non-reducing SDS-PAGE gel profiles were measured using the Carestream software program. The ratio of intensities for cross-linked bands migrating above the 70 kDa marker to the un-cross-linked monomeric band migrating at ~70 kDa was calculated for each rHA and time point and plotted as function of storage time below.

Figure 6A shows the reducing and non-reducing SDS-PAGE data obtained on days 0 and 28 of the study. Under non-reducing conditions, rHA proteins typically migrate as monomers and cross-linked oligomers which form a ladder of higher molecular weight bands. The primary full-length HA0 migrates at approximately 62 kDa, and dimer and trimer migrate at approximately 120 and 180 kDa, respectively. rHA proteins migrating above the 210 kDa marker are due to the formation of higher order cross-linked structures. The cross-linking is disulfide mediated based on the SDS-PAGE profiles obtained under reducing conditions which show predominantly monomeric rHA (Figure 2A,B and Figure 6A).

The day 0 results show essentially no disulfide mediated cross-linking by non-reducing SDS-PAGE for the 5Cys and 3Cys variants, while the wild-type and the 2Cys SDV show evidence of cross-linked dimer, trimer and higher order oligomers. After 1 month at 25°C, the 5Cys SDV shows no sign of cross-linking, and the 3Cys SDV shows a relatively small amount (approximately 15%) of cross-linked dimer and trimer compared to an extensive increase in cross-linking for the 2Cys SDV (approximately 63%) and the wild-type rHA (approximately 72%) during the same time period. A loss of intensity of HA0 (~60 kDa) is observed for both the 2Cys and the wild-type rHA as a result of the cross-linking, while the intensity of HA0 protein remains comparable on days 0 and 28 for the 3Cys and 5Cys SDVs. Thus, the decrease in cross-linking observed with the number of cysteine substitutions in the TM/CT correlates with the corresponding increase in the relative potency for these SDVs.

The intensities of the individual bands in the SDS-PAGE gels were measured at each time point in the study, and the ratio of intensities of the cross-linked forms to the un-cross-linked rHA (HA0) was determined for each rHA. These ratios were plotted as a function of time (Figure 6B). It is evident that cross-linking steadily increased for the wild-type H3 rHA and the 2Cys SDV for up to 14 days and then stabilized. A slight increase in cross-linking was observed for the 3Cys SDV on day 28, while no cross-linking was observed for the 5Cys SDV during the study period.

**Figure 7:**
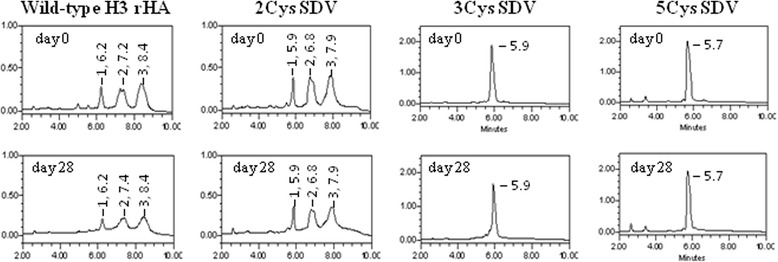
**RP-HPLC profiles for the H3 rHA proteins.** Each H3 rHA was analyzed in duplicate. Representative chromatograms from the day 0 and 28 time points are provided. rHAs were incubated with 25 mM DTT for at least 30 minutes prior to analysis. ~25 μg of each rHA was injected onto a Poros R1 column and an acetonitrile gradient applied.

The RP-HPLC profiles for the wild-type and 2Cys SDV H3 rHAs are comparable, but different from the RP-HPLC profiles for the 3Cys and 5Cys H3 SDV rHAs (Figure 7). The 3Cys and 5Cys SDV H3 rHAs elute as a single peak between 5.7-5.9 minutes, while the wild-type and 2Cys SDV H3 rHAs elute in three separate peaks (labeled 1–3) between 6–9 minutes. The elution time for the first peak is very similar for all the three SDVs (5.7-5.9 minutes), while the first peak for the wild-type elutes slightly later (6.2 minutes). Additionally, retention times for peaks 2 and 3 are slightly shorter for the 2Cys compared to the wild-type rHA. By day 28, the three peaks decrease in intensity and broaden for both the wild-type and 2Cys SDV rHA suggesting a change in the conformation of the rHA. In contrast, the profiles for the 3Cys and 5Cys SDVs showing a single peak are similar at the beginning and end of the study with one exception. A leading tail on the elution peak for the 3Cys rHA is observed on day 28, and coincides with the appearance of cross-linked rHA by SDS-PAGE on day 28 for the 3Cys SDV. These results suggest that 3Cys SDV is effective in reducing the disulfide mediated cross-linking, but all five cysteine substitutions of the 5Cys SDV are required to completely eliminate disulfide mediated cross-linking and prevent potency loss.

**Figure 8:**
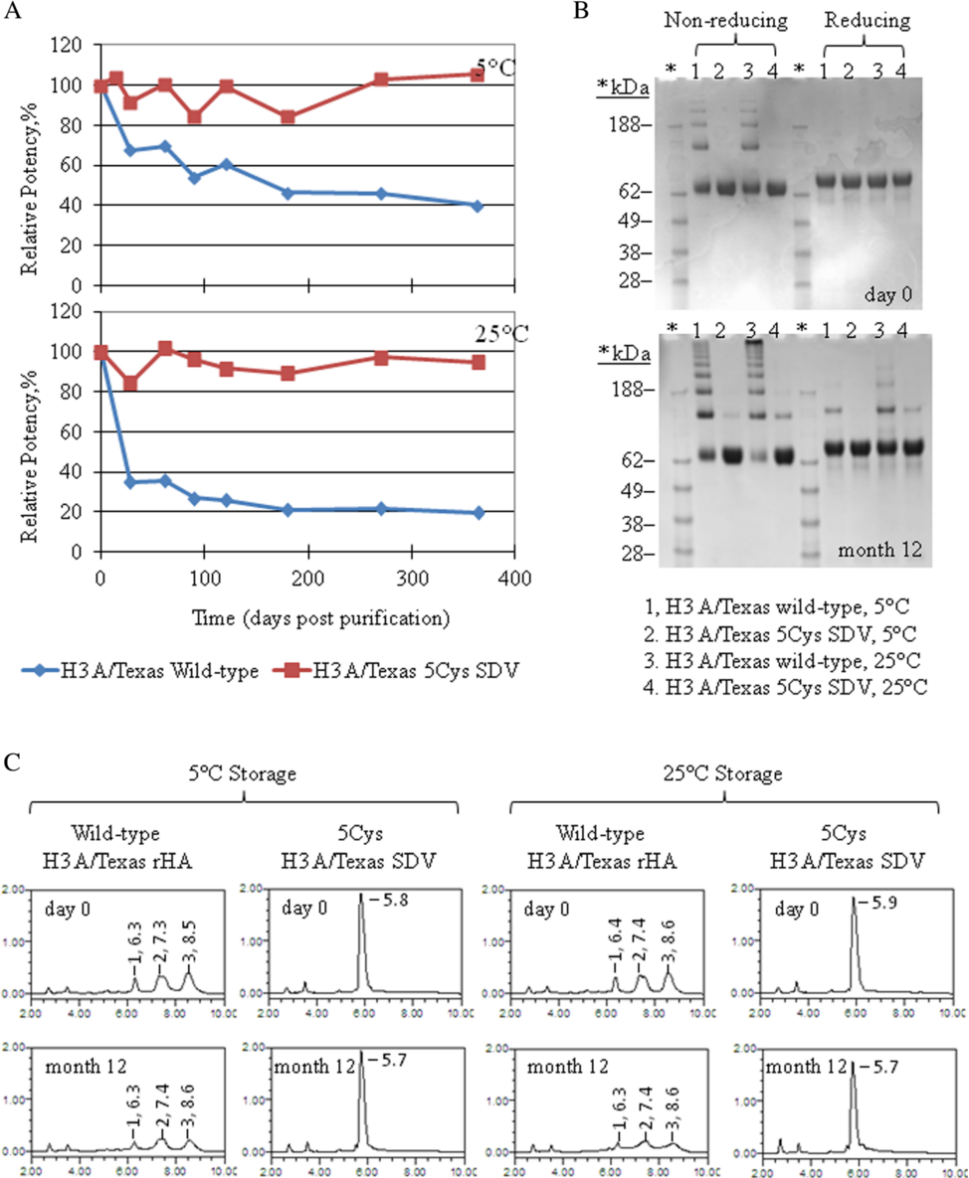
**A-C Experimental data for H3 A/Texas 5Cys SDV and wild-type rHA stored at 5°C and 25°C.** Potency by SRID, cross-linking by SDS-PAGE, and hydrophobicity by RP-HPLC were monitored monthly for 12 months. **A**. Shown are the potency results obtained at each time point for the rHA proteins stored at 5°C (top graph) and 25°C (bottom graph). For each rHA, the potency results are plotted relative to the day 0 value. **B**. Shown are the non-reducing and reducing SDS-PAGE results obtained on day 0 and after 12 months for the sets of wild-type and 5Cys SDV rHA proteins stored at 5°C and 25°C. **C**. Shown are the RP-HPLC profiles obtained on day 0 and 12 months for the sets of wild-type and 5Cys SDV rHA proteins stored at 5°C and 25°C.

The marked improvement in relative potency and the lack of disulfide mediated cross-linking for the 5Cys SDV in this study was confirmed for another 5Cys SDV rHA and corresponding wild-type rHA derived from the H3 A/Texas influenza strain. These rHA proteins were purified and stored at two temperatures, 5°C and 25°C. Potency by SRID, cross-linking by SDS-PAGE, and hydrophobicity by RP-HPLC were monitored monthly up to 12 months (Figure 8).

### Antigenicity is unaffected by cysteine substitutions in the TM and CT domains

To determine the antigenicity of the rHA proteins, the hemagglutination inhibition (HAI) assay was performed using two different polyclonal antisera. In addition, the CBER reference antigen for H3 A/Perth/16/2009 HA (lot# 70) was also included in the assay for comparison with rHA proteins. Specific binding of the antibodies to different antigenic sites in the HA proteins interferes with the ability of the HA protein to agglutinate red blood cells and forms the basis of the HAI assay. The HAI endpoint or titer, the reciprocal of the last dilution of antiserum that completely inhibits hemagglutination, was determined for each rHA protein using both antisera and compared to the CBER reference HA antigen in Table 5. Using both antisera, the HAI endpoints of the Cys SDV H3 rHA were equivalent or within 2-fold of the wild-type H3 rHA and the CBER reference antigen for H3 A/Perth HA. The results confirm that cysteine substitutions introduced into the TM and CT domain of H3 rHA did not alter the presentation of the antigenic sites.

**Table 5:** **HAI titers for wild-type and Cys SDV H3 rHAs**

**H3 HA protein**	**HAI titer**	
	**Rabbit poly clonal anti-H3 rHA Ab**	**Sheep polyclonal anti-H3 HA Ab**
Wild-type	10240	2560
2Cys	5120	2560
3Cys	5120	1280
5Cys	10240	2560
#70 H3 HA CBER	5120	2560

### Antigen-specific antibody production is unaffected by cysteine substitutions in the TM and CT domains

**Figure 9:**
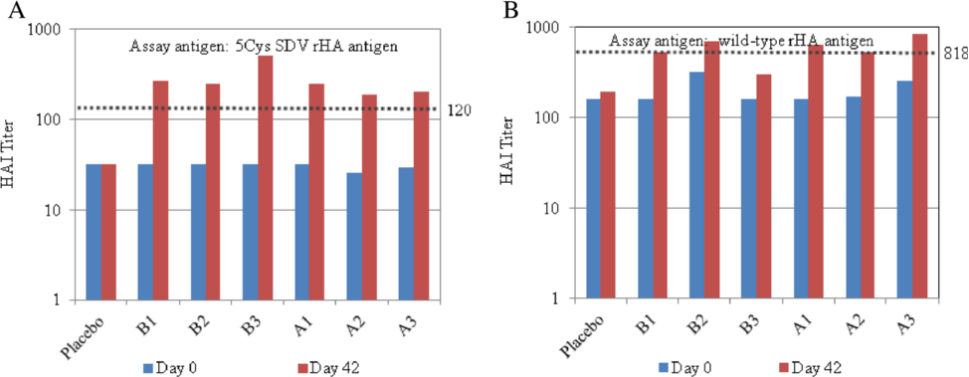
**A and B Pre-clinical mouse immunogenicity study results.** Sera samples from mice immunized with the H3 A/Texas 5Cys SDV rHA antigen (B1-B3) and with the H3 A/Texas wild-type rHA antigen (A1-A3) were tested in the HAI assay using both antigens, the H3 A/Texas 5Cys rHA antigen (Panel **A**, left) and the H3 A/Texas wild-type rHA antigen (Panel **B**, right). In each plot, the dotted line represents 4× the mean value for the day 0 titer.

A comparative study was performed in mice using the 5Cys SDV rHA and corresponding wild-type rHA from the H3 A/Texas strain. The mouse study was performed by Josman LLC and included 7 cohorts, three for each rHA antigen and one for a placebo, with 10 acclimated Balb/c mice in each cohort. On day 0 and day 21 of the study, mice were administered a 25 μL intramuscular injection of either the wild-type rHA antigen at one of three doses (1 μg, 3 μg, or 10 μg) or the corresponding 5Cys SDV antigen at one of three doses (1 μg, 3 μg, or 10 μg). The placebo group received an equal volume dose of the formulation buffer. Pre-immune serum samples collected on day 0 were tested alongside serum samples collected on day 42 in the HAI assay (see above) using both the wild-type rHA and SDV rHA as the test antigens. The number of responders having at least a 4-fold change in geometric titer on day 42 compared to day 0 were similar for all test groups receiving the two different rHA antigens regardless of the antigen used in the assay (wild-type or 5Cys SDV). Further, the average geometric mean titers for all groups were comparable regardless of the antigen and dose delivered to the mice (Figure 9). Based on the results, C-terminal cysteine substitutions and the degree of disulfide cross-linking do not impact the levels of antigen-specific antibody responses upon immunization.

## Discussion

The influenza HA protein is an integral membrane protein and the primary target for inducing neutralizing antibodies to influenza virus. When purified to homogeneity, the protein forms a rosette structure which exposes the immunogenic, trimeric ectodomain, while the transmembrane domains and short cytoplasmic tails are most likely buried inside the rosette structure [37]. It has long been demonstrated that formation of trimeric HA is not dependent on the TM or CT domains [38], though it has been suggested that the TM domain may contribute to the stability of the trimer [37]. Similarly, the early stages of HA mediated membrane fusion are unaffected by deletion of the TM and CT domains and replacement by a non-proteinaceous GPI anchor [39-42]. Therefore, the wild-type rHA and the TM and CT cysteine SDVs in this study are expected to form similar trimers and rosettes.

An evaluation of the human influenza strains which fall into 3 subtypes, H1N1 (H1), H3N2 (H3) and, B, shows that the H3 rHAs have five cysteine residues in the TM and CT domains (Figure 1A). The two cysteine residues in the CT domain are highly conserved and are found in all human influenza strains. The cysteine residue positioned at the interface between the TM and CT is found in H3 and H1 HAs, but not in B HAs. The biological role of these cysteine residues has been the subject of several studies which have identified them as sites of palmitoylation/acylation involved in membrane fusion and viral replication [25-28]. While these activities are indispensable for the virus, they are not required for expression and purification of the recombinant hemagglutinin protein during vaccine manufacture. In fact, replacing all five of the TM/CT cysteines in the 5Cys SDV or different subsets of them in the 3Cys and 2Cys SDVs (Figure 1B) result in rHA proteins having starting yields, final purities, and purified yields that are comparable to or better than the wild-type rHA protein (Table 1 and Figure 2A). In contrast, substitution of the two conserved cysteine residues in the globular head domain of the rHA protein, C64 and C76, with serine residues results in low expression further confirming that the cysteine residues in the globular head domain play a critical role in the folding of hemagglutinin protein, and cannot be substituted (Table 1).

The folding and functional activity of the wild-type rHA and the TM/CT SDVs were compared by evaluating the trypsin resistance profiles, and hemagglutination activities. Trypsin resistance is a property of properly folded trimeric HA proteins, while hemagglutination requires the organization of these trimers into higher order rosette structures that can interact with red blood cells through their sialic acid and form a lattice like network. Trypsin treatment cleaves properly folded, full-length, trimeric HA into two fragments, an amino-terminal HA1 fragment and a carboxy-terminal HA2 fragment, while denatured or monomeric rHA is digested by the endoprotease. Trypsin treatment of the wild-type and SDV rHAs results in the same characteristic HA1 and HA2 cleavage with apparent molecular weights of ~50 kDa for HA1 and ~28 kDa for HA2 (Figure 2B). Hemagglutination occurs when the red blood cells interact via their sialic acid moiety with HA rosettes, and form a lattice like network that remains in suspension. In the absence of hemagglutination, the cells do not form the lattice like network, and a halo or circle of settled cells is observed. These results demonstrate that the hemagglutination activity of SDVs of rHA is comparable to the wild-type rHA (Table 2). Further, these results suggest that the cysteine substitutions in the TM/CT domains do not impact the functional activity of H3 SDV rHAs.

Having established proper folding and function, the particle size and thermal stability of the wild-type and SDV rHA proteins were determined. The average particle size by DLS (Table 3) for the wild-type rHA and the TM/CT SDVs is in the range expected for rosettes structures. The volume mean diameter of the 3Cys and 5Cys SDVs is 35 nm and 37 nm, respectively, compared to 39 nm for the wild-type H3 rHA protein. The diameter of the particles for the 2Cys SDV is consistently 10 nm larger than the wild-type, and may reflect that the replacement of cysteine to serine at position 523 is not optimal and leads to particles of larger size. By EM (Figure 3B), the rosettes of the wild-type and SDV rHAs are strikingly similar, and indistinguishable in terms of size and appearance. In addition to particle size and morphology, the thermal stability of the rosettes in the SDV rHAs as measured by DSF is similar to that of the wild-type rHA (Table 4). The largest difference in T_m_, observed between the 5Cys SDV and the wild-type, is only 1.6°C. Decreased thermal stability has been reported previously for three H3 HA variants in which one or both of the two TM cysteine residues unique to this subtype were modified [29]. However, the thermal stability in this study was determined in a fundamentally different way on crude extracts using the hemagglutination activity assay. Further, our thermal stability data are in close agreement with previously report TM values for rosettes suggesting that both the WT and Cys SDV rHAs are similarly highly stable [43].

By SEC, all the rHA proteins have a single, primary peak with retention times differing by 0.5 minute or less. In addition, the wild-type and SDV rHAs elute before the largest standard, thyroglobulin, having an approximate molecular weight of 660 kDa (Figure 4A). These data also suggest that the rHA trimers assemble into rosettes structures, and the ability to form the rosettes is unaffected by the cysteine substitutions in the TM/CT domains. Differences in the homogeneity of the rosette population in the wild-type and 5Cys SDV rHAs are detected by UPLC SEC analysis of the Zwittergent 3–14 treated rHA (Figure 4B). The Zwittergent 3–14 surfactant efficiently converts the rosettes of the 5Cys SDV, but not of the wild-type rHA, to trimers. Due to its smaller size compared to the rosette, the rHA trimer elutes off the SEC column later and the peak is shifted. In contrast, Zwittergent 3–14 treatment of the wild-type rHA protein results in multiple peaks corresponding to rosette, trimer, and various species of intermediate sizes. Disulfide mediating cross-linking occurs readily in the wild-type rHA based on SDS-PAGE analysis (below), and the various cross-linked states of the protein result in multiple elution peaks as the Zwittergent 3–14 surfactant cannot disrupt the disulfide bonds.

Though the overall folding, thermal stability, size, and morphology are the same for the wild-type and TM/CT SDVs, differences in the formation of disulfide mediated cross-linked structures have been observed by SDS-PAGE (Figure 6A). Disulfide mediate cross-linking occurs readily at the C-terminus of the HA molecule through free cysteines located in the TM and CT domains. In the wild-type rHA and 2Cys SDV rHA, this cross-linking occurs during the purification process and is extensive. Cross-linked dimers, trimers, as well as higher order cross-linked oligomers are observed immediately following purification on day 0, albeit at a slightly reduced level in the 2Cys SDV. In the presence of reducing agent, the oligomers are converted to monomeric HA0 confirming that the cross-linking is disulfide mediated and due to the oxidation of free cysteine residues. Cross-linking of the 5Cys SDV rHA is not observed even after 28 days at room temperature, while cross-linked dimers and trimers of the 3Cys SDV rHA are first observed on the day 28 time point (SDS-PAGE gels for days 7 and 21 not shown). The non-reducing and reducing SDS-PAGE results for the 5Cys SDV and corresponding wild-type rHA from the H3 A/Texas strain further support the findings obtained with the H3 A/Perth SDVs (Figure 8). The formation of non-native disulfide bonds is prevented in H3 rHA proteins by replacing all five TM and CT cysteine residues.

Based on the SDS-PAGE results, the rHA protein is highly susceptible to oxidation through cysteines in both the TM and CT domain. The subset of cysteines modified in the 3Cys SDV (C539, C546, and C549) appears to be more susceptible to cross-linking than the subset of cysteines in the 2Cys SDV (C524 and C528). The latter two substitutions have little effect on the SDS-PAGE profile of the 2Cys SDV compared to the wild-type rHA, while the former three substitutions markedly reduce the formation of cross-linked rHA in 3Cys SDV. These results are not unexpected as the cysteines in the CT domain are more exposed at the C-terminus of the molecule. Free cysteine at this position may permit the cross-linking of monomers within a given trimer (intra-) and between monomers of different trimers (inter-).

The RP-HPLC profiles for H3 A/Perth rHAs (Figure 7) and the H3 A/Texas rHAs (Figure 8) are consistent with the non-reducing SDS-PAGE analyses. The multiple elution peaks observed for the wild-type rHAs and 2Cys SDV rHAs may be due to different cross-linked populations. Alternatively, they may due to the covalent attachment of fatty acids (palmitate and/or stearate) through thio-ester linkages involving the last three C-terminal cysteine residues present in both of these rHA molecules [26,44], but absent in the 3Cys and 5Cys SDV rHA proteins. Both types of populations, cross-linked rHA and acylated rHA, are more likely to be retained on the column due the increased hydrophobic character and elute later in the gradient as observed here in the RP-HPLC profiles for the wild-type and 2Cys SDV rHA.

Concurrent with evaluation of the cross-linking, the potency of the wild-type and SDV rHA proteins from the H3 A/Perth and H3 A/Texas strains were measured by the SRID assay (Figures 5 and 8). By this method, the relative potency is directly proportional to the number of TM/CT substitutions and inversely correlated with cross-linking. In the 5Cys SDV rHA in which all five TM/CT cysteines have been replaced, the potency relative to day 0 remains at 100% even after 12 months at room temperature for both the H3 A/Texas and H3 A/Perth 5Cys SDV rHAs, and no disulfide mediated cross-linking is observed. By comparison, the relative potency of the wild-type H3 rHAs drop approximately 75% after 4 weeks of storage at 25°C, and demonstrates extensive cross-linking. Compared to the wild-type rHAs, relative potency of the 2Cys SDV (two TM cysteine substitutions in H3 A/Perth rHA) drops to 50% during the same time (4 weeks at 25°C). Furthermore, the relative potency of the 3Cys SDV (three cysteine substitutions in the TM and CT domains of H3 A/Perth rHA), drops only 20% after 4 weeks storage at 25°C. While cross-linking is markedly reduced for the 3Cys SDV rHA, the 2Cys SDV shows only a marginal improvement over the wild-type rHA protein. These results clearly demonstrate that the substitution of cysteine residues in the CT domain has a significant impact on potency, but substituting cysteine residues in both the TM and CT domains has a synergistic effect on potency of the rHA.

**Figure 10:**
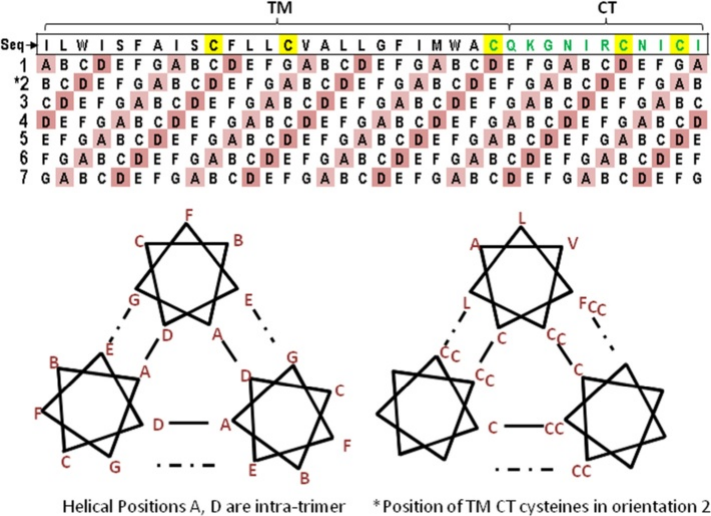
**Modeling of the TM and CT domains in the HA trimer.** The top shows the amino acid sequence of the carboxy terminus (TM and CT domains) for H3 A/Perth HA with the TM and CT cysteines highlighted in yellow (top line), and the 7 possible orientations of that sequence in a helix with the interfacial positions highlighted in pink (A and D). The schematics depict the trimer configuration (each monomer is represented by a seven pointed helical wheel) with 7 positions labeled A through G on the bottom left and one possible configuration (orientation 2) on the bottom right.

While including a reducing agent and redox buffer, STG and Citrate, in the formulation buffer has been shown to prevent an apparent potency loss associated with the formation of non-native disulfide bonds and higher order cross-linked oligomers of the HA (manuscript in preparation), the results of this study clearly show that replacing the five cysteines in the TM and CT domains with an alternative amino acid has the same effect and is a highly effective alternative solution to address potency loss. The ability of these cysteines to cross-link appears to be a direct consequence of the production and purification process that releases the TM domain from the constrained environment of the membrane bilayer and the CT domain from the reducing environment of the cell. Since the three dimensional structure of the C-terminus (TM and CT domains) has not been solved by X-ray crystallography, a theoretical structural analysis of HA using a membrane protein topology prediction algorithm was performed assuming that the transmembrane region of the HA molecule adopts and continues the alpha- helical conformation of the proceeding HA2 domain [15]. Using the program TMHMM [45,46] and the HA sequence from H3 A/Perth, seven symmetrical alpha helical trimer configurations are possible (see Figure 10). Based on this model, the TM and CT cysteines are in the correct orientation and geometry to form both intra-molecular cross-links within a trimer and inter-molecular cross-links between different trimers. Amino acids with a spacing of three or four may be found on the same face of an alpha helix and cysteine residues in those positions can form disulfide bonds between two adjacent helices, thus covalently linking helices. Cysteine residues on the outside of the helices may participate in the covalent crosslinking of higher order oligomers. The association of HA trimers through their hydrophobic transmembrane domains in rosette structures also facilitates intermolecular cross-linking by positioning the C-terminal cysteines from different trimers in close proximity to each other.

While both intra- and inter-molecular cross-linking of the HA trimer are possible, the latter may reduce diffusion, and/or antibody recognition in the SRID assay. Prior to analysis in the SRID assay, the rHA proteins are pre-treated with Zwittergent 3–14 surfactant into order to dissociate non-covalently bound rHA rosettes structures into soluble trimers. SEC-UPLC analysis of rHA before and after Zwittergent 3–14 treatment clearly shows that only a portion of the wild-type rosettes are converted to trimers; in contrast, the rosettes of the 5Cys SDV are quantitatively converted to trimers. Due to their small size (~12-14 nm in length, its longest dimension), rHA trimers can readily diffuse in to the agarose gel containing antibody and the size of the resulting precipitin ring is directly proportional to the antigen concentration. In contrast, trimers covalently bound together in rosettes through disulfide bonds are resistant to Zwittergent 3–14 pre-treatment. Depending on the extent of oxidation and/or the orientation of the cross-linked trimers, mobility of the particles through the gel may be hindered resulting in artificially small ring sizes. Alternatively, access to the epitopes may become increasingly hindered as the rosettes become covalently cross-linked through disulfide bonds resulting in a reduction in antibody binding and signal. Due to the inability of the SRID assay to accurately measure the rosette in a cross-linked state, an artificial or apparent potency loss is observed as the number of these oxidized species increases with storage of the rHA protein.

HA rosettes have been considered an indicator of immunogenicity because of their resemblance to the infectious influenza virions having HA trimeric spikes protruding from the viral envelope [36]. Additionally, the antigenic sites on the HA molecule responsible for eliciting neutralizing antibodies are located in the globular head domain approximately 135 Å away from the cysteine substitutions located at the opposite end of the elongate molecule in the TM and CT domains [9-11]. Despite this wide physical separation, structural perturbations from side chain mutations have been shown to be propagated over long distances through the peptide backbone. For this reason, the antigenic and immunological affects of C-terminal substitutions on the rHA protein were determined. The hemagglutination inhibition assay was performed using the SDV and wild-type H3 A/Perth rHA proteins in order to determine and compare recognition and binding to antibodies in sera produced using the wild-type rHA antigen. The HAI titers for the SDVs were comparable to the wild-type rHA (Table 5) indicating that the antigenic presentation of the epitopes on the SDVs has not been changed by the cysteine substitutions. Also, mice were immunized with either the H3 A/Texas 5Cys SDV or the corresponding wild-type rHA antigen. Based on similar HAI titers obtained in each group, the cysteine substitutions have no effect on the levels of rHA-specific antibody responses induced upon immunization. Thus, the cross-linked trimers in the wild-type rHA rosettes and the un-cross-linked trimers in the 5Cys SDV rHA rosettes induce comparable antibody responses in mice upon immunization. The results suggest that the potency loss observed for the wild-type rHA protein over time is largely due to the inability of the required potency assay to measure higher order cross-linked forms of the protein that retain their immunogenicity. Future work will include clinical trials to determine the impact on immunogenicity in human subjects.

In the manufacture of the rHA vaccine, the unique (non-biological) environment permits the formation of non-native disulfide bonds through free cysteines located in the TM and CT domains of the wild-type rHA protein. C-terminal cross-linking may occur to some extent in traditional split and subunit influenza vaccines as the purification process uses a detergent extraction step which may liberate the C terminus of the HA from the viral membrane. While both inter- and intra-molecular cross-linking can occur, only the latter results in cross-linked rosette structures that remain immunogenic, but are not accurately measured in the SRID assay. As a result, an apparent potency loss is observed with storage of the wid-type rHA vaccine. Cysteine mutagenesis is one way to overcome protein oxidation without the introduction of reducing agents in the formulation. However, the utility of this strategy is largely limited to the recombinant influenza vaccine since site specific mutagenesis of the virus prior to amplification in chicken cells or mammalian cells would represent a significant challenge.

## Conclusions

rHA proteins form higher-order, defined oligomers (rosettes) that become covalently cross-linked through cysteine residues in the TM and CT domains. Replacement of these cysteine residues prevents disulfide bond formation at the C-terminus, but does not impact the expression, purification, or folding of the molecule based on our work as well as others [28,29]. Moreover, the particle size and morphology, thermal stability, and antigenicity of the molecule are unaffected. Application of the cysteine mutagenesis strategy to rHA proteins derived from the other seasonal influenza subtypes (H1 and B), and pandemic strains (H5N1, H7N7) is currently underway, and a clinical trial is being planned. This technology may be extremely important for pandemic preparedness and would greatly enhance the value of the stock pile if the vaccine is not subject to potency loss at room temperature.

## Methods

### Generation of cysteine variants of rHA by site-directed substitution

The polymerase chain reaction (PCR) was used to construct three plasmid DNA constructs encoding different variants of the H3 A/Perth/16/2009 (H3 A/Perth) wild-type rHA protein. Amino acid residue changes were introduced by two complementary site directed mutagenesis (SDM) primers which contain sense mutations of the nucleotide(s). Table 6 provides the sequences of the SDM primers for the different variants with the nucleotides designed to introduce mutations in bold lower case type. The pPSC12 LIC transfer plasmid DNA, a proprietary transfer vector from PSC, containing the wild-type HA gene for the H3 A/Perth rHA protein was used as a template in the PCR for all constructs but one. The mutagenized DNA from the PCR for the triple mutation (C539A, C546A, and C549A) was used as a template in a subsequent PCR to generate the construct containing the TM and CT mutations, C539A, C546A, C549A, C524A, and C528A. The PCRs were treated with the restriction endonuclease DpnI to digest the template DNA, and transformed into *E. coli*. Plasmid DNA was isolated for sequencing. Sequencing analysis was performed using HA specific primers to confirm substitutions of the targeted amino acid residues. The PCR strategy described above was also used to substitute the five cysteine residues in the TM and CT domains of the H3 A/Texas rHA protein (C524A, C528A, C539A, C546A, C549A). The DNA containing corresponding H3 A/Texas triple cysteine substitutions (C539A, C546A, C549A) was used as the template in the PCR (Table 6).

**Table 6:** **Primers for SDV rHA proteins of H3 A/Perth and 5Cys SDV rHA of H3 A/Texas**

**Substitutions (SDV)**	**Primers (5′ to 3′)**	
**H3 A/Perth**		
C524S, C528A (Control SDV)	forward	CCTTTGCCATATCAT**c**TTTTTTGCTT**gc**TGTTGCTTTGTTGGGG
	reverse	CCCCAACAAAGCAACA**gc**AAGCAAAAAA**g**ATGATATGGCAAAGG
C539A, C546A,C549A (3Cys)	forward	GGGGTTCATCATGTGGGCC**gc**CCAAAAAGGCAACATTAGG**gc**CAACATT**gc**CATTTAAGTAAGTACCG
	reverse	CGGTACTTACTTAAATG**gc**AATGTTG**gc**CCTAATGTTGCCTTTTTGG**gc**GGCCCACATGATGAACCCC
C539A, C546A, C549A, C524A, C528A (5Cys)*	forward	CCTTTGCCATATCA**gc**TTTTTTGCTT**gc**TGTTGCTTTGTTGGGG
	reverse	CCCCAACAAAGCAACA**gc**AAGCAAAAAA**gc**TGATATGGCAAAGG
C64S,C76S (2Cys)	forward	CCTCATCAGATCCTTGATGGAAAAAACT**ct**ACACTAATAGATGCTCTATTGGGAGACCCTCAGT**c**TGATGGCTTCCAAAATAAGAAATGGG
	reverse	CCCATTTCTTATTTTGGAAGCCATCA**g**ACTGAGGGTCTCCCAATAGAGCATCTATTAGTGT**ag**AGTTTTTTCCATCAAGGATCTGATGAGG
**H3 A/Texas**		
C539A, C546A, C549A (3Cys)	forward	CATCATGTGGGCC**gc**CCAAAAGGGCAACATTAGG**gc**CAACATT**gc**CATTTGATAAGTAA
	reverse	TTACTTATCAAATG**gc**AATGTTG**gc**CCTAATGTTGCCCTTTTGG**gc**GGCCCACATGATG
C524A, C528A, C539A, C546A, C549A (5Cys)*	forward	TCCTTTGCCATATCAG**c**TTTTTTGCTT**gc**TGTTGCTTTGTTGGGG
	reverse	CCCCAACAAA**gc**AACAGCAAGCAAAAAA**g**CTGATATGGCAAAGGA

*DNA for the corresponding triple mutation (3Cys) was used as the template for the PCR reaction.

### Baculovirus generation and scale-up

The parental AcMNPV baculovirus (AcB729.3) DNA was linearized to remove the polyhedrin gene and 3′ region of ORF1629, and subsequently co-transfected with the plasmid DNA containing the rHA gene of interest into *expres*SF+ (SF+) cells. The culture was incubated for ~5 days at 27°C with shaking prior to harvesting by centrifugation. The resulting viral supernatant post transfection was used to infect a monolayer of SF+ cells to purify and isolate recombinant plaques for further scale-up. Briefly, monolayers of SF+ cells in early to mid-log phase were inoculated with serial dilutions of the transfection supernatant. A 2× Protein Sciences Formulation Medium (PSFM)/Agarose overlay was applied to the plates. After 8 days at 27°C, well isolated recombinant baculovirus plaques were identified by microscopic evaluation under low magnification and by comparison with a control of wild-type baculovirus plaques with polyhedra. Recombinant baculovirus plaques were isolated and used to infect a culture of SF+ cells. The infected culture was incubated for at least 5 days at 27°C with shaking and was harvested by centrifugation. The supernatant containing the passage 1 (P1) virus was further amplified to passage 5 virus by propagation of P1 virus through P5 in SF+ cells serum-free PSFM. The viral supernatants were harvested by centrifugation to generate the virus stocks for infection.

### Production and purification of rHA proteins

The recombinant P5 baculovirus stocks were used to produce the wild-type and variant rHA proteins. Cultures of SF+ cells (10 L) were seeded in PSFM media and maintained at 26-28°C with agitation in glass bioreactors equipped with an air overlay, and a desired dissolved oxygen concentration. After reaching a density of ~ 2.0-2.5 × 10^6^ cells/mL, the culture was infected by adding 2% (v/v) of the P5 working virus stock. The infected cultures were harvested approximately 56 hours post infection when the cell viability reached 40%-50%. The cell pellets were collected by centrifugation for further purification and analysis.

The rHA protein was solubilized from the SF+ cell membrane using Triton X-100 surfactant and released into a buffer for further purification. Cell debris and suspended solids were removed from the cell extract by depth filtration. The rHA in the filtrate stream was concentrated by ion exchange chromatography, and further purified from process related impurities using a hydrophobic interaction column followed by Q membrane filtration. Tangential flow filtration was used for final buffer exchange into phosphate buffered saline (PBS). The proteins were diluted, if necessary, to a final total protein concentration of 400–600 μg/mL. The purified rHA proteins were filtered through a 0.2 μm filter and stored at 4°C until further use.

### Stability testing and biochemical and biophysical characterization of rHAs

The purified H3 A/Perth wild-type and SDV rHA proteins were stored under accelerated conditions by placing the samples at 25°C. Aliquots were removed on days 0, 7, 14 or 21, and 28 in order to determine potency by SRID, cross-linking by SDS-PAGE, and hydrophobicity by RP-HPLC. Additional potency measurements were made at approximately 2.5, 6, 9 and 12 months. Proper folding and functional activity were assessed by the trypsin resistance and hemagglutination assays (HA), respectively. Particle size was determined by Dynamic Light Scattering (DLS) and Size Exclusion Chromatography (SEC), thermal stability by Differential Scanning Fluorimetry (DSF), and antigenicity using the hemagglutination inhibition (HAI) assay.

The purified H3A/Texas 5Cys SDV and corresponding wild-type rHA proteins were stored under both accelerated (25°C) and real time (5°C) storage conditions. These rHA proteins were monitored for potency by SRID, cross-linking by SDS-PAGE, and hydrophobicity by RP-HPLC on a monthly basis for four months.

### Total protein measurements

Total protein was determined by the bicinchoninic acid method (BCA) using the BCA protein assay kit (Cat#23225, Thermo Scientific, Rockford, IL) following the manufacturer’s recommendations.

### Non-reducing and reducing SDS-PAGE

SDS-PAGE was performed under both reducing and non-reducing conditions. The rHA samples were mixed with 2× Laemmli buffer (Bio-Rad, 161–0737), and approximately 4-5 μgs of rHA was loaded per lane. For reducing conditions, a final concentration of 100 mM DTT was added to the Laemmli-rHA solution using a 500 mM DTT stock (Pierce, product# 20291, lot# ND170603) and incubated in a 100°C heat block for 3–5 min prior to loading on to the gel. Non-reduced and reduced samples were separated using either 3-8% Tris-Acetate gels (Life Technologies, EA03752BOX) and Tris-Acetate SDS Running Buffer (Invitrogen, LA0041) or 4-12% NuPAGE Bis-Tris Gels (Cat# NP0323, Life Technologies Corporation, Carlsbad, CA) and 1× MES Running Buffer (50 mM MES, 50 mM Tris, 0.1% sodium dodecyl sulfate, 1 mM EDTA pH 7.3) at 150 volts for 1 hour. Protein bands were visualized by Coomassie staining (Bio-Rad, 161–0787). Gels were stained for 1 hour followed by a water wash until protein bands developed. Alternatively, purity and trypsin resistance gels were stained for 1 hour in 0.1% Brilliant Blue R, 7.7 M reagent alcohol, 1.75 M glacial acetic acid, and de-stained in 10% acetic acid. Gels were scanned using Gel Logic 212 Pro Imaging System, and densitometry was performed using the Carestream Molecular Imaging software (Carestream Health, Incorporated, New Haven, CT).

### Trypsin resistance assay (TR)

Purified rHA proteins at a total protein concentration of 250 μg/mL were analyzed both with and without heat denaturation (5 minutes at 100°C) for trypsin resistance. The samples were incubated in the presence or absence of 50 μg/mL trypsin (Cat#T1426, Sigma) for 30 min at 2-8°C. Digestion was stopped by incubating the samples in 2× disruption buffer (120 mM Tris pH 6.8, 20% glycerol, 4% sodium dodecyl sulfate, 0.2% bromophenol blue, 200 mM dithiothreitol) in a 100°C heat block for 3–5 minutes. Samples were analyzed by SDS-PAGE as described above.

### Hemagglutination assay (HA)

The hemagglutination assay was performed in 96 well u-bottom microtiter plates (Corning Inc., Part# 2797). Each monovalent bulk batch was loaded at a total protein concentration of 1 μg/mL in the first lane, and two-fold serial dilutions were performed in 1 × PBS across the plate. An equal volume of fresh, washed guinea pig red blood cells (RBCs) (Lampire Biological Labs, Cat# 7243108) at a concentration of 0.5% in 1 × PBS was added to each well. After approximately 1 hour of incubation at room temperature, the plates were scored for agglutination of red blood cells in presence of rHA. The HA activity of the purified rHA is defined by the dilution at which partial agglutination was observed (i.e., 50% of the RBCs were agglutinated or the pellet appeared loose). If only fully agglutinated and/or tight pellets were observed, the endpoint was defined as the average of the dilutions at which agglutinated and tight pellets were observed.

### Size exclusion chromatography (SEC)

Size exclusion chromatography (SEC) was performed on a Waters Alliance 2695 HPLC system (SEC-HPLC) with a PDA detector using a Biosuite 450, 8 μm HR SEC column (7.8 × 300 mm) (Waters; Cat#186002166) or a Waters H-Class Bio UPLC system (SEC-UPLC) with PDA detector, using an acuity BEH 450 chromatography column. The latter system was used to analyze select rHAs including the wild-type and 5Cys SDV before and after pre-incubation with 1% Zwittergent 3–14, the surfactant used to pre-treat rHA samples for the SRID potency assay. For all SEC analyses, the mobile phase consisted of 1 × PBS pH 7.2 with 300 mM NaCl and a flow rate of 0.25 mL/min was used. Approximately 17.5 μg of rHA was injected per sample. Columns were calibrated by analyzing the proteins in a gel filtration HMW calibration kit (Cat# 28-4038-42, GE Healthcare Piscataway, NJ), and a standard curve was generated. The data were analyzed using the Empower 2 Chromatography Data Software (Waters Corporation, Milford, MA).

### Dynamic light scattering (DLS)

Dynamic Light Scattering was performed using a Malvern Zetasizer Nano-S (Malvern Instruments, Worcestershire, UK) according to the manufacturer’s instructions. Zetasizer software (Version 6.20) was used for data analysis. Each purified rHA protein was analyzed undiluted at a total protein concentration between 400–600 μg/mL. A 70 μL aliquot of each rHA was dispensed into a micro-cuvette and measured in duplicate. Each measurement consisted of 12–14 individual scans. The volume-average size distributions from each set of scans were averaged for a given rHA.

### Electron microscopy (EM)

Negative staining scanning electron microscopy was performed by Paragon Bioservices. Samples were prepared using a continuous carbon grid method, using 400-mesh copper grids with a nitrocellulose film. The rHA proteins were diluted 5-fold in water, applied to the grid, and immediately stained with uranyl formate solution (1%). Electron microscopy was performed at room temperature. Ten images were obtained for each rHA at a magnification of 135,000 × .

### Differential scanning fluorimetry (DSF)

DSF was performed using a fluorescent dye (ProteoStat, Enzo Life Sciences) and a real time PCR machine (Applied Biosystems 7500 Fast Real-Time PCR System). Samples were scanned from 25°C to 99°C, and the fluorescence intensity was monitored as a function of temperature. The temperature where rHA unfolded (characterized by a transition in the fluorescence signal) was determined from the second derivative plot, and expressed as midpoint transition temperature (T_m_). The T_m_ is the temp where 50% of the molecules are in the unfolded state. The data were analyzed using the Applied Biosystems Protein Thermal Shift Software (Version 1.1).

### Reverse phase (RP) HPLC analysis

RP-HPLC was performed using a polystyrene POROS R1/10 (2.1 mm × 100 mm) column (Applied Biosystems, 1-1012-16) on a Waters Alliance 2695 HPLC system with a PDA detector. Mobile phase A consisted of 0.1% trifluoroacetic acid, 5% acetonitrile in water (Solvent A), and mobile phase B consisted of 0.1% trifluoroacetic acid in acetonitrile (Solvent B). The column was pre-equilibrated in 20% B. The following mobile phase gradient was applied to each sample: 2 min 20-40% B, 1 min 40% B, 7.5 min 40-100% B, and 3.5 min 100% B. A flow-rate of 0.8 mL/min was used and the absorbance was monitored at 214 nm. To prevent carry-over between samples, the column was washed after each sample by injecting 1% Zwittergent (100 μL) on to the column and applying the following wash gradient: 100-20% B, 8 minutes. The rHA samples were pre-treated for 30 minutes with 25 mM DTT using a 500 mM DTT stock, and were filtered using 0.45 μm syringe filter units (Millipore, SLVR04NL) prior to RP-HPLC analysis using Empower 2 Chromatography Data Software (Waters Corporation, Milford, MA). Each sample was analyzed in duplicate using approximately 20 μg of rHA per injection.

### In vitro potency measurements

The rHA potency was determined by the SRID assay as described previously [19,20]. Briefly, rabbit polyclonal antiserum for the wild-type H3 A/Perth rHA antigen was added to melted 1% agarose (Cat# 50010, SeaKemME, Lonza, Rockland, ME ) in 1 × PBS (pH 7.2) (Cat# 20012–050, Life Technologies Corporation) at 54-56°C and allowed to solidify at ambient temperature on GelBond film (Cat# 53734, Lonza, Rockland, ME). Wells of 4 mm in diameter were punched into the gels and serial dilutions of both the reference standard of known potency, and the purified rHA antigens were loaded. Reference and sample proteins were incubated with Zwittergent 3–14 (Cat# 693017, Calbiochem, Darmstadt, Germany) at a final concentration of 1% for at least 30 minutes prior to preparing the serial dilutions in 1 × PBS containing 1% Zwittergent 3–14. The gels were placed in a sealed moist chamber at room temperature for ~18 hours. Following incubation, the gels were washed first with 1 × PBS (pH 7.2) and then with water. The gels were allowed to dry prior to staining with Coomassie Brilliant Blue R250 (Cat#BO149, Sigma). The gels were then destained and dried again for analysis. The diameters of the precipitin rings were measured in two orthogonal directions using the GT Vision SRID Reading Program (GT Vision LLC, Hagerstown, MD). Recombinant HA potency was calculated in μg/mL by the parallel line bioassay method using reference and test rHA antigen response curves (log antigen dilution vs. log zone diameter). Statistical parameters for determining test validity were based on correlation coefficients (r ≥ 0.95) and the equality of slopes (t < 4.604) between test and reference antigens.

### Hemagglutination inhibition (HAI)

The HAI assay was performed in 96-well u-bottom microtiter plates. Each rHA antigen was standardized to have an HA titer of 4 HA units/25 μL (this titer provides four wells of hemagglutination activity). The standardized preparations of diluted rHA were then mixed with 2-fold serially diluted antibody. Mouse serum samples were pre-treated with receptor destroying enzyme (RDE (II) “SEIKEN”, Denka Seiken Co., LTD., Japan) prior to testing to remove non-specific binding of the rHA antigen. The antigen and antibody were allowed to incubate for approximately 15 minutes prior to the addition of an equal volume of 0.5% guinea pig red blood cells. Plates were incubated at ambient temperature for approximately 45 minutes to 1 hour, the time required for the cells to completely settle in the absence of any hemagglutinating activity (negative control lane) in PBS buffer. The HAI titer, the reciprocal of the last dilution of antiserum that completely inhibits hemagglutination, was determined. The assay was performed using two polyclonal antisera: rabbit polyclonal anti-H3 antibody generated against BEVS derived wild-type H3 A/Perth rHA and sheep polyclonal anti-H3 antibody generated against bromelain cleaved HA derived from the H3/Victoria/361/2011 influenza virus propagated in eggs. A reference antigen from Center for Biologics Evaluation and Research (CBER) for H3 A/Perth/16/2009 HA (lot# 70) was included in the assay for comparison to the rHA proteins.

### Mouse study

The antigen formulations were shipped to Josman LLC for immunization of acclimated Balb/c mice (10 mice per cohort). The mice received a 25 μL intramuscular injections on day 0 and day 21. The mice in the control antigen cohorts, A1, A2, and A3, received 1, 3, and 10 μg of the wild-type H3 A/Texas rHA. The mice in the test antigen cohorts, B1, B2, and B3, received 1, 3, and 10 μg of the H3 A/Texas 5Cys SDV rHA. The mice in the placebo cohort C were administered a 25 μL intramuscular injection of formulation buffer. Blood (~250 μL) was collected from each mouse before the first injection on day 0 and at the end of the study on day 42. The blood was allowed to clot for approximately 2 hours at ambient temperature prior to centrifuging to isolate the sera in the supernatant. The sera samples were stored frozen at −20°C until testing in the HAI assay. This study was performed under protocol JLP-005.010 by Josman LLC, a licensed research facility (USDA license number 93-R-0260; OLAW-NIH/PHS Assurance license number A3404-01) that adheres to guidelines set forth through the Animal Welfare Act (7 U.S.C. 2131 et seq.), and through the Office of Laboratory Animal Welfare of the National Institutes of Health.
